# A Patient With CAD Deficiency Responsive to Uridine and Literature Review

**DOI:** 10.3389/fneur.2020.00064

**Published:** 2020-02-05

**Authors:** Ling Zhou, Han Xu, Tianshuang Wang, Ye Wu

**Affiliations:** Department of Pediatrics, Peking University First Hospital, Beijing, China

**Keywords:** CAD, developmental delay, epilepsy, anemia, uridine

## Abstract

CAD encodes a multifunctional enzyme involved in *de novo* pyrimidine biosynthesis, and pyrimidine can be alternatively recycled from uridine. Trio whole-exome sequencing identified CAD compound heterozygous mutations in a new male patient with global developmental delay (DD), refractory epilepsy, and anemia with anisopoikilocytosis. We further reviewed all published cases with CAD deficiency. Five patients were collected from two publications, including three males and two females, and all presented with DD, drug-resistant epilepsy, and anemia with anisopoikilocytosis. Four out of six patients (including the present case) were supplemented with uridine, which led to immediate cessation of seizures, resolved anemia with anisopoikilocytosis, and progress in global development. The other two patients, who were not treated with uridine, died at the ages of 4 and 5 years. In summary, CAD deficiency is probably a treatable neurometabolic disorder.

## Introduction

A trifunctional protein encoded by *CAD* contains three highly conserved enzymatic activities, carbamoyl-phosphate synthetase 2 (CPS2), aspartate transcarbamylase (ATCase) and dihydroorotase (DHOase), and is involved in *de novo* pyrimidine biosynthesis (1–3). Alternatively, pyrimidines can be recycled from uridine (4). In 2015, the first patient with compound heterozygous mutations in *CAD* was reported. This phenotype was related to early infantile epileptic encephalopathy-50 (EIEE50, MIM#114010), and this defect could be rescued with uridine (1). Only five patients have been reported worldwide thus far (1, 4). Three out of five patients were supplemented with uridine, which was effective. We report a new patient with compound heterozygous mutations in *CAD* who presented with developmental delay, refractory epilepsy, and anemia with anisopoikilocytosis. Supplementation with oral uridine led to dramatic improvement in the clinical symptoms.

## Case Report

**Figure 1:**
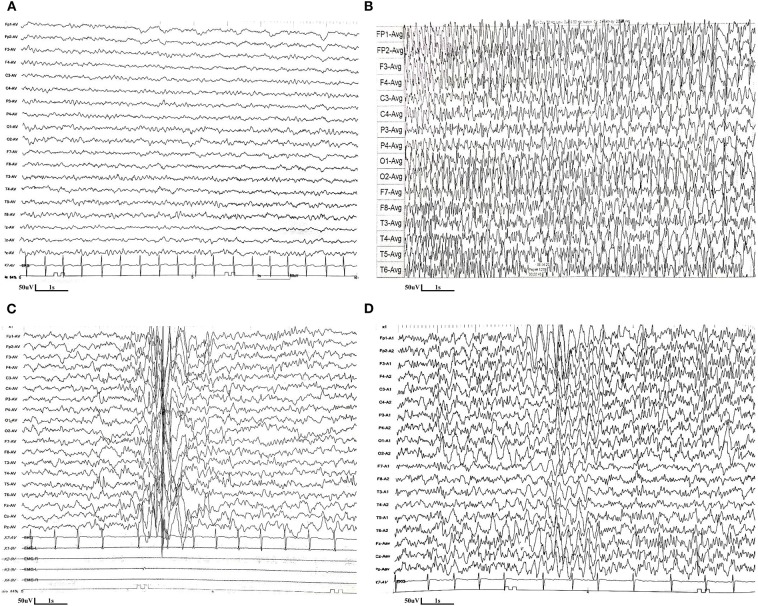
EEG features of the patient with CAD deficiency. **(A)** The first EEG performed at 1.5 years old was normal. **(B)** EEG performed at 2.5 years old revealed the presence of absence seizures, with the ictal EEG as 3 Hz generalized spike-and-wave discharges. **(C)** The suspicious epileptic myoclonus was recorded during EEG monitoring at 4.5 years old. **(D)** EEG performed at 5.3 years old (5 months after uridine treatment) showed poor background, with spike and spike-slow waves in the bilateral parieto-central region during sleep.

The patient is a boy aged 5.5 years old, the second child of a healthy non-consanguineous couple. His sister, who showed developmental delay, anemia, and epilepsy, died at the age of seven in 2014 without definite diagnosis. The boy was born at term after normal pregnancy. The developmental milestones before 1 year of age were generally normal. He could walk without support and speak monosyllables at 1 year old. At 1.5 years old, he started to present with focal epilepsy, and his development in cognitive and motor function showed gradual regression. He had reduced visual acuity and was diagnosed with strabismus at 2 years old. The boy could barely walk independently and respond to verbal commands at 4.5 years old. The first EEG performed at 1.5 years old was normal (Figure 1A). Levetiracetam was started. There was a seizure-free period that lasted for 8 months. EEG performed at 2.5 years old revealed the presence of absence seizures, with the ictal EEG as 3 Hz generalized spike-and-wave discharges (Figure 1B). The suspicious epileptic myoclonus was recorded during EEG monitoring at 4.5 years old (Figure 1C). Although several antiepileptic drugs (levetiracetam, lamotrigine, clonazepam, and valproic acid) were attempted, the frequency of epileptic seizures gradually increased. He had seizures almost daily after the age of four.

**Figure 2:**
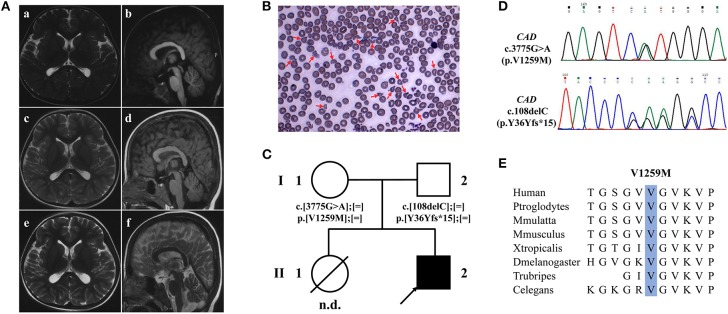
Brain MRI, peripheral blood smear and WES findings of the patient with CAD deficiency. **(A)** Brain MRI of the patient, T2-weighted axial (a,c,e,f) and T1-weighted sagittal (b,d). Initial brain MRI performed at 2.5 years old was unremarkable (a,b) but showed progressive global cerebral and cerebellar atrophy at 3.6 (c,d) and 5 years old (e,f). **(B)** Peripheral blood smear showed abnormal erythrocytes with characteristics of varying sizes and abnormal shapes. **(C)** Family pedigree of the patient. Squares denote males, circles females, solid symbols affected persons, and slashes deceased persons. **(D)** Two variants in *CAD*, c.108delC (p.Y36Yfs*15) and c.3775G>A (p.Val1259Met), were identified by WES and validated by Sanger sequencing. **(E)** Val1259 of *CAD* is evolutionally conserved among different species.

The first brain magnetic resonance imaging (MRI) performed at 2.5 years old was unremarkable. Progressive cerebral and cerebellar atrophy was shown at 3.6 and 5 years old (Figure 2A). Routine blood tests indicated moderate anemia (hemoglobin 7.1 g/dl, reference 11–16 g/dl; mean corpuscular volume 70.8 fl; reference range 80–100 fl) at 8 months and thereafter. Peripheral blood smear showed abnormal erythrocytes manifested as varying sizes and abnormal shapes, suggestive of anisopoikilocytosis (Figure 2B). Blood biochemical tests and metabolic profiles were unrevealing.

Trio whole-exome sequencing (WES) was performed at 5 years old. Compound heterozygote variants were identified in the proband of the family in *CAD* (Figure 2C), which were c.108delC (p.Tyr36Tyrfs^*^15) and c.3775G>A (p.Val1259Met) (Figure 2D). Both variants were interpreted as likely pathogenic on the basis of standards of the American College of Medical Genetics. The amino acid Val1259 is highly conserved from humans to *C. elegans* (Figure 2E).

Oral uridine (Jarrow) was started for a total of 100 mg/kg/day at 5 years of age. Supplementation with uridine led to cessation of seizures starting on the second day after treatment, and no seizures were observed over a follow-up period of 7 months. Antiepileptic drugs were gradually withdrawn, and now he is only taking lamotrigine and levetiracetam, which are still under dosage reduction. Moreover, the boy showed significant improvement in global development; he is now able to walk and run and communicate with complicated sentences. EEG performed 5 months after uridine treatment showed poor background, with spike and spike-slow waves in the bilateral parieto-central region during sleep (Figure 1D). Three months after the treatment, blood smears were normalized, and anemia resolved (hemoglobin 13.5 g/dl; mean corpuscular volume 83.8 fl).

## Literature Review

Case reports on CAD deficiency were searched from PubMed (www.ncbi.nlm.nih.gov/pubmed), and the search deadline was July 5, 2019. The following data were collected: history in the neonatal period, developmental milestones, familial history, epileptic seizures, neuroimaging, EEG, anemia, mutations in *CAD*, and response to uridine treatment.

Five patients from four different families were collected from two publications, including three males and two females (1, 4). The clinical features are summarized in Table 1. All the patients presented with epilepsy, mostly generalized seizures, which occurred at a median age of 1.5 years old (6 months−2 years old). In addition, they all showed significant global development delay and even retrograde motor and cognitive development. All the patients manifested anemia with anisopoikilocytosis, with a median age at onset of 1.4 years old (1.0–2.8). The brain MRI showed that the brain volume was initially normal, followed by progressive cerebral and cerebellar atrophy in several years.

**Table 1:** Clinical characteristics of patients with CAD deficiency.

**References**	**Gender**	**Current age/age at death**	**Family history**	**Variations in *CAD***	**DD**	**Age of seizure onset**	**Anisopoikilocytosis/amanemia**	**EEG**	**Brain MRI**	**Treatment with uridine (follow-up period)**	**Seizure free**	**Anisopoikilocytosis resolved**
Present study	Male	5.4 years	+	c.108delC, p.Tyr36Tyrfs*15 c.3775G>A, p.Val1259Met	+ (<1 year)	1.5 years	+/+	Generalized	GA	+ (6 months)	+	+
Ng et al. (1)	Male	4 years	–	c.1843-1G>A, p.? c.6071G>A, p.Arg2024Gln	+ (<1 month)	17 months	+/+	n/r	n/r	+ (n/r)	n/r	n/r
Koch. et al. (4)	Male	4 years (deceased)	+	c.98T>G, p.Met33Arg c.98T>G, p.Met33Arg	+ (<1 year)	20 months	+/+	n/r	GA	–	–	–
Koch. et al. (4)	Female	3.5 years	+	c.98T>G, p.Met33Arg c.98T>G, p.Met33Arg	+ (<1 year)	2 years	+/+	Multifocal	n/a	+ (7 months)	+	+
Koch. et al. (4)	Male	5 years (deceased)	–	c.98T>G, p.Met33Arg c.98T>G, p.Met33Arg	+ (<18 months)	2 years	+/+	n/r	GA	–	–	–
Koch. et al. (4)	Female	5.4 years	–	c.1843-3C>T, p.? c.5365C>T, p.Arg1789*	+ (<4 months)	6 months	+/+	Encephalopathic pattern+ multifocal	GA	+ (5 months)	+	+

*DD, developmental delay; GA, global cerebral and cerebellar atrophy; n/r, not reported; n/a, not available*.

Three out five patients were supplemented with oral uridine. The exact curative effect in a patient reported by Ng et al. was not described in detail (1). For the other two patients reported by Koch et al., when uridine supplementation was started at 5.2 years of age in one patient, she was bedridden, and in a minimally conscious state, seizures occurred every second day. During the 5 months of follow-up, two very short self-limiting seizures were observed, and she made obvious development in her cognition and movement (4). Treatment in the other patient led to immediate cessation of seizures in a few days. Moreover, anisopoikilocytosis was resolved within 2–3 months. The other two patients, who were not treated with uridine, died at the ages of 4 and 5 years.

## Discussion

With the newly reported case, there are now six cases carrying biallelic mutations in *CAD* worldwide. Seven mutations were identified in *CAD* among the six patients, including three missense, one frameshift, one non-sense and two splicing mutations. CAD plays a vital role in the *de novo* synthesis of pyrimidine biosynthesis (1, 5, 6), and pyrimidines can alternatively be recycled from uridine, which is essential in the process of protein glycosylation, lipid metabolism, and polysaccharide biosynthesis (7, 8). Previously reported mutations were predicted to result in altered tertiary protein configuration or deteriorated protein function (4).

Developmental delay, drug-resistant epilepsy and anemia with anisopoikilocytosis were key characteristics presented in all patients. The cases showed regression of cognitive and motor function. Seizures were refractory to antiepileptic drugs. Anemia was prominent in patients with CAD deficiency but usually mild or moderate. A peripheral blood smear revealed abnormal erythrocytes with characteristics of varying sizes and abnormal shapes (anisopoikilocytosis), acanthocytes, and schistocytes resulting in a dyserythropoietic anemia phenotype (4). Bailey et al. suggested that dyserythropoiesis might be due to a shortage of pyrimidine-dependent nucleotide-lipid cofactors required for erythrocyte membrane synthesis (9).

The neuroimaging features were non-specific. Progressive supra- and infratentorial atrophy was observed in four patients with brain MRI available. Other biomarkers, such as urine purines and pyrimidines (including uracil, uric acid, hypoxanthine, or xanthine), were normal. Urine purine testing showed only a mild elevation of guanosine. Urine orotic acid testing was usually normal.

The natural disease course could be lethal. Patients who received uridine supplementation showed obvious benefit, manifested as immediate cessation of seizures, resolved anisopoikilocytosis, and improved development. Therefore, CAD deficiency could be considered a treatable inborn error of metabolism with refractory epilepsy.

## Conclusion

We reported a new patient with biallelic *CAD* mutations who was characterized by developmental delay and regression, refractory epilepsy and anemia with anisopoikilocytosis. Uridine supplementation dramatically rescued these abnormalities. CAD deficiency could be considered a treatable inborn error of metabolism.

## Data Availability Statement

Datasets can be found here: https://github.com/2Ling-Zhou/frontiers-in-neurology.

## Ethics Statement

The studies involving human participants were reviewed and approved by the clinical research ethics committee of Peking University First Hospital. Written informed consent to participate in this study was provided by the participants' legal guardian/next of kin. Written informed consent was obtained from the individual(s), and minor(s)' legal guardian/next of kin, for the publication of any potentially identifiable images or data included in this article.

## Author Contributions

LZ drafted/revised the manuscript and accepted responsibility for conducting the research and final approval. LZ, HX, and TW acquired the data and accepted responsibility for conducting the research and final approval. YW drafted/revised the manuscript and acquired the data and accepted responsibility for conducting the research and final approval.

### Conflict of Interest

The authors declare that the research was conducted in the absence of any commercial or financial relationships that could be construed as a potential conflict of interest.
